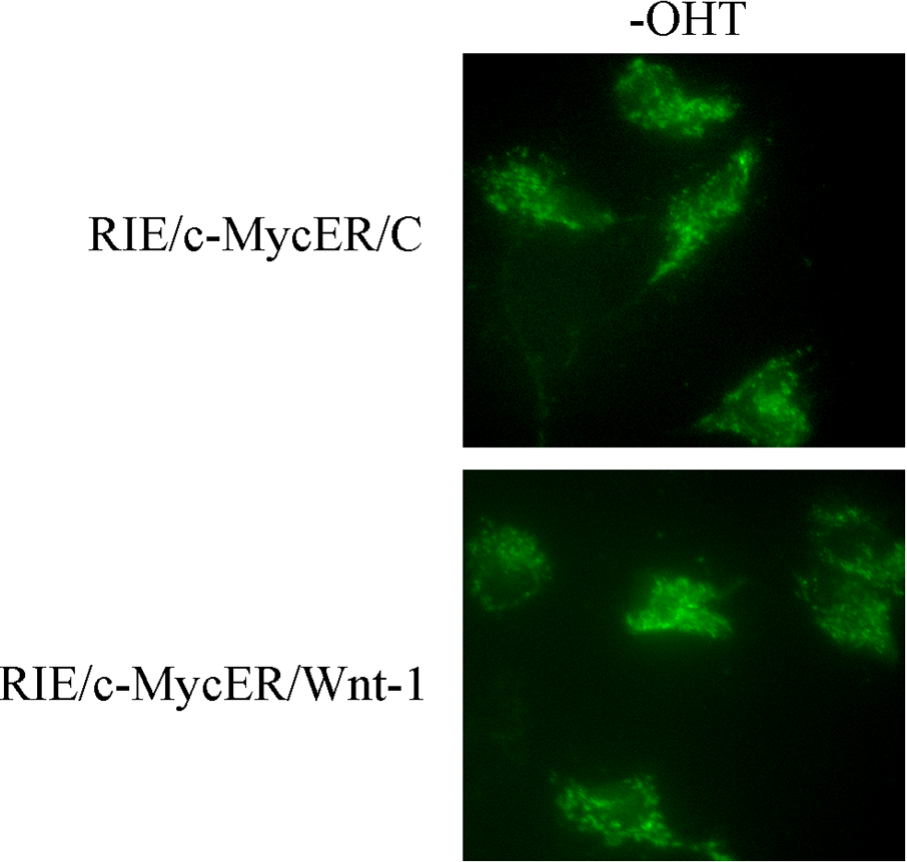# Correction: Wnt signaling promotes oncogenic transformation by inhibiting c-Myc–induced apoptosis

**DOI:** 10.1083/jcb.20020111004052021c

**Published:** 2021-04-08

**Authors:** Zongbing You, Daniel Saims, Shaoqiong Chen, Zhaocheng Zhang, Denis C. Guttridge, Kun-liang Guan, Ormond A. MacDougald, Anthony M.C. Brown, Gerard Evan, Jan Kitajewski, Cun-Yu Wang

Vol. 157, No. 3 | 10.1083/jcb.200201110 | April 29, 2002

The authors report an error in Figure 3 A of the original publication. The image of RIE/c-MycER/C (– OHT) cells was accidentally duplicated and used to also describe the RIE/c-MycER/Wnt-1 (– OHT) cells. Given the elapsed time since the data were first generated, the authors are no longer able to locate the original data images. Instead, they have repeated the experiments in question using the same cells and find that, consistent with the original results, both cells show punctuate staining for cytochrome c in the absence of OHT. The revised panels are shown here. The interpretation of the figure and the conclusions of the manuscript are unaffected.

**Figure:**